# Isolated branched-chain amino acid intake and muscle protein synthesis in humans: a biochemical review

**DOI:** 10.31744/einstein_journal/2019RB4898

**Published:** 2019-08-30

**Authors:** Carina de Sousa Santos, Fabrício Expedito Lopes Nascimento

**Affiliations:** 1 Universidade Federal dos Vales do Jequitinhonha e Mucuri, Diamantina, MG, Brazil.; 2 Instituto de Pesquisas, Ensino e Gestão em Saúde, Belo Horizonte, MG, Brazil.

**Keywords:** Leucine, Valine, Isoleucine, Amino acids, branched-chain, Hypertrophy, Muscle, skeletal

## Abstract

Alongside a proper diet, ergogenic aids with potential direct and/or indirect physical performance enhancing effects are sought after for improved adaptation to physical training. Nutritional ergogenics include diet composition changes and/or dietary supplementation. Branched-chain amino acids valine, leucine and isoleucine are widely popular among products with ergogenic claims. Their major marketing appeal derives from allegations that branched-chain amino acids intake combined with resistance physical exercise stimulates muscle protein synthesis. Evidence supporting the efficacy of branched-chain amino acids alone for muscle hypertrophy in humans is somewhat equivocal. This brief review describes physiological and biochemical mechanisms underpinning the effects of complete protein source and branched-chain amino acid intake on skeletal muscle growth in the postabsorptive and post-exercise state. Evidence in favor of or against potential anabolic effects of isolated branched-chain amino acid intake on muscle protein synthesis in humans is also examined.

## INTRODUCTION

Alongside proper diets, ergogenic aids with potential direct and/or indirect physical performance enhancing effects are widely sought after for improved adaptation to physical training. These aids are thought to boost energy production and use, support recovery from exercise, improve body composition and increase resistance to peripheral and central fatigue. Nutritional ergogenic aids include diet composition changes and/or dietary supplementation.^1^

According to the International Society of Sports Nutrition (ISSN), ergogenic supplement claims must be backed up by plausible data and supported by solid evidence of efficacy; these supplements must also be legal and safe.^2^

Branched-chain amino acids (BCAA) enjoy great popularity among allegedly ergogenic supplements. The marketing appeal of these products derives from claims that isolated BCAA intake combined with resistance physical exercise stimulates muscle protein synthesis. This long-standing (over 35 years) claim is based on cellular and animal model studies reporting enhanced anabolic intracellular signaling in response to BCAA intake.^3^ However, evidence supporting the efficacy of isolated BCAA intake for muscle hypertrophy in humans is equivocal.

This study set out to review muscle protein synthesis in the postabsorptive state and after resistance physical exercise from a basic biochemistry perspective, and to examine existing evidence in favor of or against claims of potential anabolic effects of isolated BCAA intake on muscle protein synthesis in humans. PubMed^®^, Latin American and Caribbean on Health Sciences Literature (LILACS) and Scientific Electronic Library Online (SciELO) electronic databases were searched using the following Medical Subject Headings (MeSH) terms: “leucine”, “valine”, “isoleucine”, “branched-chain amino acids”, “muscle protein” and “exercise”. A secondary search was conducted based on the references listed in selected articles.

### Muscle protein synthesis

Proteins consist of 20 amino acids (AA) linked by peptide bonds and arranged in different combinations and amounts. Nine out of these 20 AA are thought to be essential (EAA - essential amino acids), *i.e* ., not synthesized in the body and necessarily obtained through diet. The BCAA leucine, isoleucine and valine account for almost 50% of muscle protein EAA. The remaining 11 EAA are classified as nonessential (NEAA, nonessential amino acids), as they can be synthesized in the body.^4 , 5^

In healthy individuals with normal mobility, dynamic balance between protein degradation and synthesis orchestrates skeletal muscle protein maintenance. In the postabsortive ( *i.e* ., fasting) state, muscle protein degradation exceeds synthesis, leading to net protein loss. In the postprandial state, synthesis exceeds degradation, since intake of some nutrients, such as proteins and carbohydrates, stimulates muscle protein synthesis and insulin release, suppressing degradation.^6^ Therefore, muscle hypertrophy requires a positive net protein balance ( *i.e* ., muscle protein synthesis in excess of muscle protein degradation).

Physical exercise and nutrient availability are the major drivers of muscle protein synthesis in adult individuals.^7 , 8^ The anabolic effects of nutrients are boosted primarily by transfer and incorporation of AA obtained through the diet into skeletal muscle proteins.^7^ These effects are particularly associated with EAA.^9^

The full range of EAA and the 11 NEAA must be present in proper amounts for muscle protein synthesis. Therefore, muscle protein synthesis is limited by lack or low availability of any of the EAA, whereas lack of NEAA can be offset by increased *de novo* synthesis.^3^

In the postprandial state, within approximately 30 to 45 minutes of consumption of a protein-rich meal (average time required for digestion, absorption and transport of AA to the systemic circulation), EAA availability increases and muscle protein synthesis rates exceed muscle protein degradation rates, inducing an anabolic state that peaks between 1.5 and 3 hours after meal.^10^

Aminoacidemia-induced muscle protein synthesis is transient. In the postabsortive state ( *i.e* ., 2 to 3 hours after meal), plasma EAA levels drop below postprandial levels if no more dietary protein is consumed. In these circumstances, plasma EAA level maintenance (and hence protein turnover) relies on protein breakdown in skeletal muscles, the major body protein reservoir.^11^ The impact of factors such as protein amount and quality, protein intake distribution throughout day and physical exercise on the balance between protein degradation and synthesis must be emphasized.

In humans, muscle protein degradation exceeds synthesis by approximately 30% in the postabsorptive state, since 25% of EAA released into the plasma are captured by other tissues and 5% are oxidized in the muscle. The remaining 70% are reutilized for sustained muscle protein synthesis.^12^ Hence, muscle protein degradation always exceeds muscle protein synthesis in the postabsorptive state due to muscle protein catabolism and catabolic conditions determined by lack of dietary EAA intake.

### BCAA and muscle protein synthesis in the postabsorptive state

The hypothesis that isolated BCAA intake in the postabsorptive state optimizes muscle protein synthesis is based on the premise that exogenous intake decreases the 30% degradation rate by increasing EAA availability for synthesis – instead of oxidation or release into the plasma.^3^ This is thought to be partly due to the AA leucine, as leucine alone is able to induce a muscle protein synthesis response via activation of the mechanistic target of rapamycin complex 1 (mTORC1), a vital cell growth regulator.^13^

Louard et al.,^14 , 15^ tested this hypothesis in humans submitted to overnight fasting. In their study, the effects of intravenous BCAA infusion for 3 and 16 hours on muscle protein synthesis and degradation were investigated. Both infusion protocols increased plasma BCAA levels, whereas plasma levels of other EAA decreased. Muscle protein degradation and synthesis also decreased by the same degree, but the synthesis/degradation balance remained negative. The fact that muscle protein degradation was mitigated by isolated intake of three out of 11 EAA, but remained higher than muscle protein synthesis, suggested the catabolic state prevailed in order to release other EAA required for synthesis. It seems therefore plausible to assume BCAA intake alone cannot create an anabolic state leading to muscle protein synthesis in excess of degradation, at least in theory.

Another important question is whether anabolic pathway activation and increased muscle protein synthesis are separate events. Rising insulin levels are a potent anabolic signaling pathway activator, but are not associated with enhanced muscle protein synthesis in the absence of EAA.^16^ In contrast, intake of small amounts (3g) of EAA stimulates synthesis regardless of anabolic signaling pathway activation.^17^ Should muscle protein synthesis be limited by activation of factors triggering pathway initiating, increases in plasma EAA levels, however small, would not have this effect. These findings demonstrated muscle protein synthesis in humans is limited by availability of the full range of EAA rather than anabolic signaling pathway activation.^3^

### BCAA and post-exercise muscle protein synthesis

Combination of physical exercise (resistance physical exercise in particular) with protein intake maximizes and prolongs muscle protein synthesis stimulation for approximately 24 hours post-workout due to increased tissue sensitivity to anabolic properties of AA.^8 , 18 , 19^

The effects of leucine on human muscle protein synthesis were investigated by Churchward-Venne et al.^20^ Participants of that study (young, fit men) consumed 25g of whey protein enriched with 3g of leucine (enough to induce maximal muscle protein synthesis stimulation after resistance physical exercise)^21^ or one quarter of that dose after one resistance physical exercise session. Suboptimal and optimal doses of leucine-enriched whey protein similarly increased muscle protein synthesis for 1 to 3 hours post-workout; however, only the optimal (25g) whey protein dose was able to sustain increased synthesis up to 5 hours.

The muscle protein synthesis enhancing potential of higher leucine doses was investigated by the same research group.^22^ Participants of that study (young, fit men) consumed leucine-enriched whey protein after one session of resistance physical exercise, as follows: 25g of whey protein containing 3g of leucine; one quarter of that whey protein dose containing 0.75g, 3g or 5g of leucine, or 5g leucine plus similar amounts of isoleucine and valine (3g of each). All treatments equally increased muscle protein synthesis for 1.5 hours post-workout. However, only the suboptimal dose of whey protein containing 5g of leucine was as effective as 25g of whey protein in sustaining increased muscle protein synthesis for 4.5 hours post resistance physical exercise.

Potential reasons why the suboptimal whey protein dose containing 5g of leucine and high amounts of valine and isoleucine failed to sustain muscle protein synthesis were also investigated. It was argued that BCAA may compete for the same carrier in intestinal and muscle cells.^23 , 24^ Therefore, addition of the other two BCAA may have limited sustained muscle protein synthesis due to lower leucine uptake by cells, as suggested by lower blood and intracellular leucine levels in the first hour post-intake in participants receiving this treatment.

Leucine apparently stimulates muscle protein synthesis after resistance physical exercise; however, as with other BCAA, the full range of EAA must be available in levels that will not promote competition for cell carriers or limit their action.

Muscle protein synthesis in response to isolated intake of 5.6g of BCAA or placebo following resistance physical exercise was recently investigated in humans (young, fit men). Within 4 hours, muscle protein synthesis was 22% higher in the treated group as compared to the placebo group.^25^ In spite of statistical significance, this increase was six times lower compared to muscle protein synthesis of similar duration in response to consumption of whey protein containing similar amounts of BCAA and EAA.^20 , 22 , 26^

### International organization recommendations

The International Olympic Committee makes no specific recommendations regarding BCAA intake. In contrast, the ISSN does not recommend the use of BCAA to maximize muscle protein synthesis given the limited evidence and inconsistent results in favor of efficacy.^2 , 27 , 28^

Resistance physical exercise increases muscle tissue sensitivity to AA for up to 24 hours post-exercise, regardless of whether protein is consumed prior to, during or 1 to 3 hours post-workout. The major factor is evenly spread protein intake throughout the day, according to individual recommendations. Incremental induction of an anabolic state by repeated resistance physical exercise and diet is what boosts skeletal muscle remodeling and hypertrophy.^29 , 30^

For this reason, both organizations support recommendations of combined resistance physical exercise and daily consumption of 1.6g to 2.2g/kg of body mass of complete, high quality EAA-rich protein containing 700 to 3.000mg of leucine in evenly distributed amounts throughout the day (every 3 to 4 hours) for maximal muscle protein synthesis, including one dose after exercise and one close to bed time.^2 , 27 , 28^

## CONCLUSION

Existing evidence suggests BCAA stimulate muscle protein synthesis following resistance physical exercise. However, in the absence of other essential amino acids, BCAA are not able to sustain maximal synthesis responses. Lack of sustained muscle protein synthesis stimulation equals poor physiologic value. Therefore, a growing body of literature emphasizes that BCAA supplementation alone does not enhance muscle protein synthesis any more than consumption of complete, high quality protein containing the full range of essential amino acids.
