# Supplementary material for: Isolated branched-chain amino acid intake and muscle protein synthesis in humans: a biochemical review
Source: Einstein (Sao Paulo). 2019 Aug 30;17(3):eRB4898. doi: 10.31744/einstein_journal/2019RB4898 (PMC6718193; doi:10.31744/einstein_journal/2019RB4898)
Supplement: Translation

# Translation: Consumo isolado de aminoácidos de cadeia ramificada e síntese de proteína muscular em humanos: uma revisão bioquímica

**Authors:** Carina de Sousa Santos, Fabrício Expedito Lopes Nascimento

**Affiliations:** 1 Brasil Universidade Federal dos Vales do Jequitinhonha e Mucuri, Diamantina, MG, Brasil.; 2 Brasil Instituto de Pesquisas, Ensino e Gestão em Saúde, Belo Horizonte, MG, Brasil.

**Keywords:** Leucina, Valina, Isoleucina, Aminoácidos de cadeia ramificada, Hipertrofia, Músculo esquelético

## Abstract

No treinamento físico, buscam-se, além de uma dieta adequada, recursos ergogênicos que possam maximizar direta e/ou indiretamente o desempenho físico. Entre as categorias de recursos ergogênicos, o nutricional compreende a modulação da composição dietética e/ou uso de suplementação. A comercialização dos suplementos de aminoácidos de cadeia ramificada valina, leucina e isoleucina possui muita popularidade entre aqueles com alegação ergogênica. O principal marketing está na afirmação de que o consumo isolado de aminoácidos de cadeia ramificada associado ao exercício físico resistido estimula a síntese de proteína muscular. As evidências da eficácia da ingestão isolada de aminoácidos de cadeia ramificada para a hipertrofia muscular em humanos parecem equivocadas. Nesta breve revisão, apresentamos a compreensão fisiológica e bioquímica de como a ingestão de uma fonte completa de proteína e de aminoácidos de cadeia ramificada afeta o crescimento do músculo esquelético no estado pós-absortivo e pós-exercício. Mostramos também as evidências que suportam ou não a afirmação dos potenciais efeitos anabólicos na síntese de proteína muscular dos aminoácidos de cadeia ramificada quando consumidos isoladamente em humanos.

## INTRODUÇÃO

Para aprimorar as adaptações do treinamento físico, buscam-se, além de uma dieta adequada, recursos ergogênicos, que possam, direta e/ou indiretamente, maximizar o desempenho físico, por meio da produção e eficiência da utilização de energia, da recuperação das sessões de exercício, da melhora da composição corporal e da maior resistência à fadiga periférica e central. Entre as categorias de recursos ergogênicos, o nutricional compreende a modulação da composição dietética e/ou uso de suplementação.^1^

A *International Society of Sports Nutrition* (ISSN) considera um suplemento como ergogênico quando a ciência básica por trás da sua alegação possui lógica teórica; é legal e seguro, e há um número grande de evidências sólidas que apoiam e suportam a eficácia de alegação ergogênica.^2^

A comercialização dos aminoácidos de cadeia ramificada (BCAA - *branched-chain amino acids* ) possui muita popularidade entre os suplementos com alegação ergogênica. O principal marketing está na afirmação de que o consumo isolado de BCAA associado ao exercício físico resistido estimula a síntese de proteína muscular. A alegação de que a síntese de proteína muscular é estimulada pelos BCAA tem mais de 35 anos, tendo sido baseada em estudos em modelo celular e animal, que demonstraram aumento da sinalização intracelular de vias anabólicas em resposta a estes.^3^ No entanto, as evidências da eficácia da ingestão isolada de BCAA para a hipertrofia muscular em humanos são equivocadas.

Assim, da perspectiva da bioquímica básica, o objetivo deste trabalho é revisar a síntese de proteína muscular, no estado pós-absortivo e pós-exercício físico resistido, e as evidências que suportam ou não a afirmação dos potenciais efeitos anabólicos dos BCAA na síntese, quando consumidos isoladamente em humanos. Foi conduzida uma busca eletrônica nas bases de dados PubMed^®^, Literatura Latino-Americana e do Caribe em Ciências da Saúde (LILACS) e *Scientific Electronic Library Online* (SciELO) usando os termos *Medical Subject Headings* (MeSH) “ *leucine* ”, *“valine”, “isoleucine”, “branched-chain amino acids”, “muscle protein”* e *“exercise”.* Uma pesquisa secundária foi realizada por meio da leitura das listas de referência dos artigos.

### Síntese de proteína muscular

As proteínas são formadas por 20 aminoácidos (AA) unidos por ligações peptídicas que se combinam em diferentes formas e quantidades. Dos 20 AA, 9 são considerados AA essenciais (EAA - *essential amino acids* ), o que significa que não podem ser sintetizados pelo corpo, devendo ser obtidos pela dieta. Os BCAA leucina, isoleucina e valina são três dos nova EAA e representam quase 50% do total de EAA em proteínas musculares. Os 11 AA restantes são considerados não essenciais (NEAA - *nonessential amino acids* ), pois podem ser sintetizados no corpo.^4 , 5^

Em indivíduos saudáveis e com apropriada mobilidade, as proteínas do músculo esquelético são mantidas por um equilíbrio dinâmico entre degradação e síntese de novas proteínas. No estado pós-absortivo (jejum), a degradação de proteína muscular excede a síntese, levando à perda líquida de proteínas. No estado pós-prandial, a síntese excede a degradação, pois a ingestão de alguns nutrientes, como proteínas e carboidratos, estimula a síntese de proteína muscular e aumenta a liberação de insulina, que suprime a degradação.^6^ Assim, a hipertrofia muscular só acontece quando o balanço líquido de proteínas for positivo (quando a síntese de proteína muscular exceder a degradação de proteína muscular).

Os dois principais determinantes da síntese de proteína muscular em indivíduos adultos são o exercício físico e a disponibilidade de nutrientes.^7 , 8^ Os efeitos anabólicos dos nutrientes são impulsionados principalmente pela transferência e pela incorporação de AA obtidos das proteínas da dieta, em proteínas do músculo esquelético.^7^ Mais precisamente, estes efeitos são atribuíveis aos EAA.^9^

Para a síntese de novas proteínas musculares, todos os EAA, juntamente dos 11 NEAA, devem estar presentes em quantidades adequadas. A síntese de proteína muscular está limitada pela falta/baixa disponibilidade de qualquer EAA, ao passo que a escassez de NEAA pode ser compensada pelo aumento da produção de novo.^3^

No estado pós-prandial, aproximadamente entre 30 e 45 minutos após a ingestão de uma refeição contendo proteína (tempo médio para digestão, absorção e transporte de AA para a circulação sistêmica), a disponibilidade de EAA é aumentada, e a taxa de síntese de proteína muscular excede a de degradação de proteína muscular, produzindo um estado anabólico e atingindo um máximo entre 1,5 a 3 horas.^10^

A ativação da síntese de proteína muscular induzida por aminoacidemia é transitória. No estado pós-absortivo, após 2 a 3 horas da última refeição, se não houver nova ingestão dietética de proteína, as concentrações plasmáticas de EAA caem abaixo dos valores pós-prandiais. Nesta circunstância, a disponibilidade plasmática contínua de EAA, por conseguinte o *turnover* proteico, é mantida às custas do catabolismo das proteínas do maior reservatório corporal: a musculatura esquelética.^11^ Importante ressaltar que o balanço descrito pode ser modificado diante de fatores como quantidade e qualidade da proteína, distribuição do consumo de proteínas ao longo do dia e exercício físico.

Em humanos, no estado pós-absortivo, a degradação de proteína muscular excede a síntese em torno de 30%, devido ao fato de que 25% dos EAA são liberados no plasma e captados por outros tecidos, e 5% são utilizados para oxidação dentro do próprio músculo. O restante, aproximadamente 70%, é utilizado novamente para manter a síntese de proteína muscular.^12^ Então, no estado pós-absortivo, a taxa de síntese sempre será menor que a de degradação de proteína muscular devido ao catabolismo das proteínas musculares, e um estado anabólico não pode ocorrer na ausência de ingestão dietética de EAA.

### BCAA e síntese de proteína muscular no estado pós-absortivo

No estado pós-absortivo, a hipótese de que os BCAA isoladamente poderiam otimizar a síntese de proteína muscular está baseada no fato de que a ingestão exógena diminuiria os 30% de degradação, ao disponibilizar mais EAA para síntese − ao invés de estes serem desviados para oxidação ou liberados no plasma.^3^ Parte de toda esta popularidade é devida ao AA leucina, que, por si só, é capaz de exercer uma resposta na síntese de proteína muscular, ao ativar o complexo 1 da proteína-alvo mecanística da rapamicina (mTORC1 - *mechanistic target of rapamycin complex* 1), considerado um regulador fundamental do crescimento celular.^13^

O grupo de Louard et al.,^14 , 15^ testou esta hipótese. Os pesquisadores avaliaram em humanos, após uma noite de jejum, a infusão intravenosa de BCAA por 3 e por 16 horas, na resposta de síntese e de degradação de proteína muscular. Em ambos os protocolos de tempo, a infusão aumentou as concentrações plasmáticas de todos os três BCAA, enquanto as concentrações de outros EAA diminuíram. Também em ambos, a degradação de proteína muscular foi reduzida em quantidade similar à redução na síntese, mas o equilíbrio entre síntese e degradação continuou negativo. Assim, quando apenas três dos EAA foram consumidos isoladamente, a degradação de proteína muscular foi até amenizada, mas ainda permaneceu acima da síntese de proteína muscular, significando que o estado catabólico predominou para disponibilizar os outros EAA necessários para a síntese. Desse modo, é teoricamente impossível, para o consumo de apenas BCAA, criar um estado anabólico no qual a síntese exceda a degradação de proteína muscular.

Outro conhecimento importante é saber que a ativação de vias anabólicas de proteína e o aumento da síntese de proteína muscular são eventos dissociados. Um aumento na concentração de insulina é um potente ativador das vias de sinalização anabólicas proteicas, mas isso não aumenta a síntese de proteína muscular quando há deficiência de EAA.^16^ Por outro lado, o consumo de uma pequena quantidade (3g) de EAA estimula a síntese independentemente da ativação das vias de sinalização anabólicas.^17^ Este aumento nas concentrações plasmáticas de EAA, mesmo que pequeno, não teria efeito se a síntese de proteína muscular fosse limitada pelo estado de ativação dos fatores de iniciação da via. Portanto, estes resultados demonstram que o fator limitante da síntese em humanos é a disponibilidade de todos os EAA, em oposição à ativação das vias de sinalização anabólicas.^3^

### BCAA e síntese de proteína muscular pós-exercício

O exercício físico, com ênfase no tipo resistido, combinado com a ingestão de proteínas, maximiza e prolonga a estimulação da síntese de proteína muscular devido a uma sensibilidade aumentada do tecido às propriedades anabólicas dos AA por aproximadamente 24 horas após a sessão.^8 , 18 , 19^

Churchward-Venne et al.,^20^ investigaram em humanos o impacto da leucina na síntese de proteína muscular. Os participantes (homens, jovens e treinados) consumiram, após uma sessão de exercício físico resistido: uma dose de 25g de proteína do soro do leite contendo 3 g de leucina (dose suficiente para induzir estimulação máxima de síntese de proteína muscular após exercício físico resistido^21^ ou um quarto da dose de proteína do soro do leite enriquecida com 3 g de leucina. O estudo demonstrou que, ao longo de 1 a 3 horas após o exercício, a dose subótima de proteína do soro do leite enriquecida com leucina aumentou a síntese de proteína muscular similarmente à dose de 25g de proteína do soro do leite. No entanto, apenas a dose de 25g de proteína do soro do leite foi capaz de sustentar elevada a síntese por até 5 horas após o exercício.

O mesmo grupo de pesquisa conduziu outro estudo para investigar o potencial de uma maior quantidade de leucina em aumentar a síntese de proteína muscular.^22^ Os participantes (homens, jovens e treinados) consumiram, após uma sessão de exercício físico resistido, uma dose de 25g de proteína do soro do leite contendo 3g de leucina; um quarto da dose de proteína do soro do leite contendo 0,75g de leucina; um quarto da dose de proteína do soro do leite enriquecida com 3g ou 5g de leucina; um quarto da dose de proteína do soro do leite com 5g de leucina mais isoleucina e valina na mesma proporção da dose de 25g de proteína do soro do leite. Ao longo de 1,5 hora após o exercício, todos os tratamentos aumentaram a síntese de proteína muscular igualmente. No entanto, ao longo de 4,5 horas após o exercício físico resistido, somente a dose subótima de proteína do soro do leite com 5g de leucina foi tão eficaz quanto a de 25g de proteína do soro do leite em sustentar elevada a síntese de proteína muscular.

Os autores especularam o motivo de a dose de um quarto de proteína do soro do leite com 5g de leucina, valina e isoleucina também aumentadas não ter sustentado a síntese de proteína muscular. Associaram ao possível fato de que os BCAA competem pelo mesmo transportador nas células intestinais e musculares.^23 , 24^ Portanto, a adição dos outros dois BCAA pode ter limitado a sustentação da síntese de proteína muscular, devido à redução da entrada de leucina na célula, endossada pelo fato de que os participantes que receberam este tratamento apresentaram menor concentração de leucina sanguínea e intracelular na primeira hora após a ingestão.

A leucina parece estimular a síntese de proteína muscular após o exercício físico resistido, mas está sujeita ao mesmo problema que os BCAA: exige que todos os outros EAA estejam disponíveis e em quantidades que não compitam pelos transportadores celulares e nem limitem sua ação.

Em humanos (homens, jovens e treinados), recentemente, foi investigada a resposta de síntese de proteína muscular à ingestão isolada de 5,6g de BCAA comparada à ingestão de placebo após sessão de exercício físico resistido. Após 4 horas, a síntese foi 22% maior que o grupo placebo.^25^ Embora significativo, esse aumento foi seis vezes abaixo da resposta de síntese de proteína muscular, que é comumente sustentada pela mesma duração após a ingestão de proteína do soro do leite que contém quantidade semelhante de BCAA e demais EAA.^20 , 22 , 26^

### Recomendações de organizações internacionais

O Comitê Olímpico Internacional não faz declaração sobre a ingestão de BCAA. Já a ISSN não recomenda o uso de BCAA para maximizar a síntese de proteína muscular devido ao conjunto de evidências científicas disponíveis serem limitadas e sem resultados consistentes para apoiar a eficácia.^2 , 27 , 28^

Posto que o exercício físico resistido aumenta após a sessão, por até 24 horas, a sensibilidade do tecido muscular à ação dos AA, pouco importa a ingestão de proteínas antes, durante ou 1 a 3 horas após o exercício. O aspecto mais importante é a ingestão periódica de proteína ao longo do dia, de acordo com as recomendações individuais. É a estimulação cumulativa do estado anabólico proteico, por meio de repetidas sessões de exercício físico resistido e alimentação, que impulsiona a remodelação e a hipertrofia do músculo esquelético.^29 , 30^

Por este motivo, ambas as organizações endossam e recomendam para maximizar a síntese de proteína muscular associada ao exercício físico resistido a ingestão de fontes de proteínas completas e de alta qualidade (ricas em EAA, sendo 700 a 3.000mg de leucina) na quantidade de 1,6g a 2,2g de proteína/kg de massa corporal/dia, com as doses distribuídas uniformemente ao longo dia (a cada 3 a 4 horas), sendo uma das doses após a sessão de exercício e a outra próxima ao dormir.^2 , 27 , 28^

## CONCLUSÃO

Com base nas evidências disponíveis, os BCAA exibem capacidade de estimular a síntese de proteína muscular após o exercício físico resistido, mas, na ausência dos demais aminoácidos essenciais, não conseguem sustentar uma resposta máxima de síntese. Se a estimulação da síntese de proteína muscular não pode ser sustentada, há pouco significado fisiológico. Por esta razão, um crescente corpo de literatura continua a endossar que a suplementação isolada de BCAA em humanos não fornece benefícios adicionais à síntese de proteína muscular em detrimento à ingestão de uma fonte de proteína completa de alta qualidade, que fornece todos os aminoácidos essenciais necessários.
